# From Cell and Organismal Biology to Drugs

**DOI:** 10.1371/journal.ppat.1005002

**Published:** 2015-06-25

**Authors:** Kasturi Haldar

**Affiliations:** 1 Boler-Parseghian Center for Rare and Neglected Diseases, University of Notre Dame, Notre Dame, Indiana, United States of America; 2 Department of Biological Sciences, University of Notre Dame, Notre Dame, Indiana, United States of America

**Image 1:**
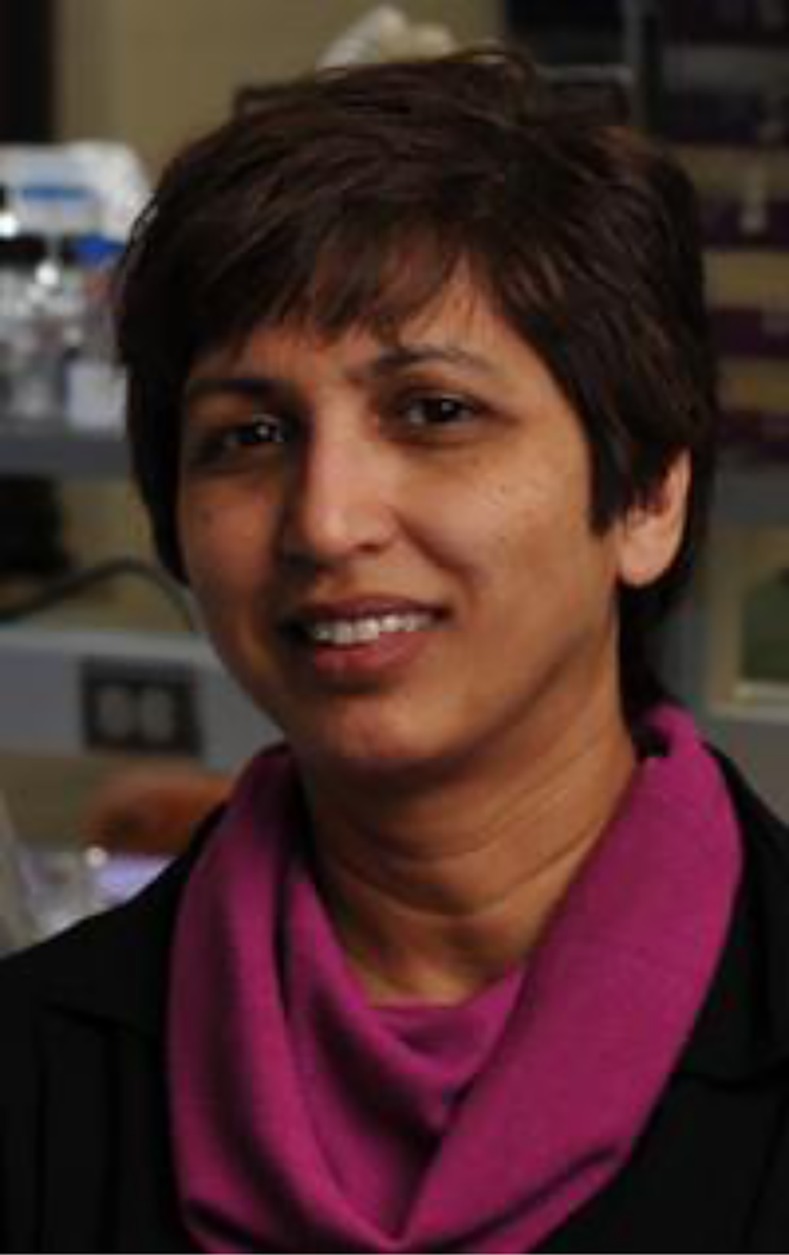
Kasturi Haldar.

Because malaria takes a major toll on human health globally, the focus on how to treat it and reduce the malaria burden is understandable. But I became interested in studying the malaria parasite because of its intracellular niche in the mature red blood cell. This is a specialized host cell designed to carry oxygen to tissues, explaining its high hemoglobin content (which also confers the red color) and deformability needed to travel through the smallest of capillaries. But these cells are dead ends, without a nucleus and many cellular capacities (such as synthesis of new nucleic acids, proteins and lipids, and nutrient uptake) that pathogens need from their host cells. Also, the mature red blood cell does not internalize or reorganize its surface membrane, a process critical for host infection by most microbes. No self-respecting virus would infect a red blood cell and most bacteria, parasites, and fungi do not. And yet malaria parasites are large pathogens (~1–2 micron) that infect a relatively small-diameter dead end host cell (~7 micron). You don’t have to be a rocket scientist to infer that the parasite must play an active role in the infection process because the host cell couldn’t—but how did it happen?

I decided that secretion from the parasite to the red cell must be very important and my lab would broadly study the parasite’s Golgi structure, because in most cells, this compartment makes important decisions on what cellular cargo is secreted. Moreover, while the parasite needed to make these secretion decisions for destinations bounded by its own surface membrane as well as those beyond in the red cell, there was no evidence of Golgi “stacks” (characteristic of this organelle) in this pathogen.

The first three years for my lab in the early 1990s were a hard slog, with a pile of negative results, because most molecular Golgi functions found in other cells are absent in the blood stage malaria parasite. Just as deep anxiety began to set in that this was the wrong question to ask, the onion began to unpeel productively. We discovered that the parasite was not just secreting cargo to the red blood cell; it was also releasing an entire subcompartment of its rather rudimentary Golgi. There has been no looking back since then, because the parasite commits a large proportion of its genetic composition to secreting and remodeling the red cell—a fact that became a certainty after 2002 when the first malaria parasite genome was sequenced. This new understanding didn’t immediately eliminate or eradicate malaria, but it accelerated the linking of fundamental biology and mechanisms of pathogenesis in labs like mine to the discovery of new therapeutic strategies (since the parasite is increasingly drug resistant). It also eventually resulted in the establishment of collaborations with major pharmaceutical companies, e.g., Eli Lilly & Co, and private product partnerships, e.g., the Medicines for Malaria Venture.

Our recent work on rare genetic disorders was initiated almost 20 years after we began our studies in malaria. But here again, we started in basic discovery, using mouse models of disease. Driven by the invariably fatal outcome that faces the small community of patients struck by the rare neurological disease, Niemann-Pick, type C (NPC), and armed with genomics, we repurposed existing drugs to improve the treatment of this disease. This effort has led to the establishment of a small pharmaceutical company (NP-C Therapeutics, LLC), the goal of which is to develop treatments for NPC and other neurological disorders, because monogenetic rare disorders, such as NPC, provide powerful portals into the more prevalent multigenic disorders, such as Alzheimer’s and Parkinson’s diseases. This approach may also provide insights into the recent outbreak and spread of Ebola, because the virus uses the NPC1 protein to infect host cells (and deficiency in NPC1 protects against infection).

Not every scientific discovery from basic research ensures translation into a therapy. But there have been more therapies realized from basic research than by the emerging trends of directed translational engagement in the absence of evidence-based research. Investment in a broad range of basic research (because it is important to query scientific problems in many ways) enables collective preparedness for new translational challenges that defy political agendas and fearmongering for partisan gain. This outcome is compelling justification for national and international agencies to prioritize unfettered discovery and basic research. Failure to do this will jeopardize future employment, training, and education at the university, college, and high school levels. Therefore, development of a new generation of researchers, trained in a virtuous cycle of rigorous scientific query and hard work, is imperative to sustain our overarching expectations for this upcoming century.

